# Association of B‐cell lymphoma 2/microRNA‐497 gene expression ratio score with metastasis in patients with colorectal cancer: A propensity‐matched cohort analysis

**DOI:** 10.1002/jcla.24227

**Published:** 2022-01-07

**Authors:** Shahad W. Kattan, Yahya H. Hobani, Nouf Abubakr Babteen, Saleh A. Alghamdi, Eman A. Toraih, Afaf T Ibrahiem, Manal S. Fawzy, Salwa Faisal

**Affiliations:** ^1^ Department of Medical Laboratory College of Applied Medical Sciences Taibah University Yanbu Saudi Arabia; ^2^ Medical Laboratory Technology College of Applied Medical Sciences Jazan University Jazan Saudi Arabia; ^3^ Department of Biochemistry College of Science University of Jeddah Jeddah Saudi Arabia; ^4^ Medical Genetics Clinical Laboratory Department College of Applied Medical Sciences Taif University Taif Saudi Arabia; ^5^ Department of Surgery School of Medicine Tulane University New Orleans Louisiana USA; ^6^ Genetics Unit Department of Histology and Cell Biology Faculty of Medicine Suez Canal University Ismailia Egypt; ^7^ Department of Pathology Faculty of Medicine Northern Border University Arar Saudi Arabia; ^8^ Department of Pathology Faculty of Medicine Mansoura University Mansoura Egypt; ^9^ Department of Medical Biochemistry and Molecular Biology Faculty of Medicine Suez Canal University Ismailia Egypt; ^10^ Department of Biochemistry Faculty of Medicine Northern Border University Arar Saudi Arabia

**Keywords:** *BCL2*, colorectal cancer, gene expression, metastasis, miR‐497, Real‐Time qPCR

## Abstract

**Background:**

Deregulated microRNAs (miRs) significantly impact cancer development and progression. Our *in silico* analysis revealed that miR‐497 and its target gene B‐cell lymphoma‐2 (*BCL2*) could be related to poor cancer outcomes.

**Purpose:**

To investigate the *BCL2*/miRNA‐497 expression ratio in colorectal cancer (CRC) and explore its association with the clinicopathological characteristics and CRC prognosis.

**Methods:**

Archived samples from 106 CRC patients were enrolled. MiR‐497 and *BCL2* gene expressions were detected by Taq‐Man Real‐Time quantitative polymerase chain reaction in propensity‐matched metastatic and nonmetastatic cohorts after elimination of confounder bias.

**Results:**

B‐cell lymphoma‐2 gene was upregulated in metastatic samples (median = 1.16, 95%CI = 1.09–1.60) compared to nonmetastatic (median = 1.02, 95%CI = 0.89–1.25, *p* < 0.001). In contrast, lower levels of miR‐495 were detected in specimens with distant metastasis (median = 0.05, 95%CI = 0.04–0.20) than nonmetastatic samples (median = 0.54, 95%CI = 0.47–0.58, *p* < 0.001). Estimated *BCL2*/miR‐497 ratio yielded a significant differential expression between the two cohort groups. Higher scores were observed in metastasis group (median = 1.39, 95%CI = 0.9–1.51) than nonmetastatic patients (median = 0.29, 95%CI = 0.19–0.39, *p* < 0.001). Receiver operating characteristic curve analysis showed *BCL2*/miR‐497 ratio score to have the highest predictive accuracy for metastasis at presentation. The area under the curve was 0.90 (95%CI = 0.839–0.964, *p* < 0.001) at cut‐off of >0.525, with high sensitivity 81.1% (95%CI = 68.6%–89.4%) and specificity 92.5% (95%CI = 82.1%–97.0%). Also, the ratio score was negatively correlated with disease‐free survival (*r* = −0.676, *p* < 0.001) and overall survival times (*r* = −0.650, *p* < 0.001). Kaplan–Meier curves showed lower survival rates in cohorts with high‐score compared to low‐score patients.

**Conclusion:**

The *BCL2*/miR497 expression ratio is associated with poor CRC prognosis in terms of metastasis and short survival.

## Graphical abstract

The workflow of explored *BCL2*/miR‐497 prognostic utility.

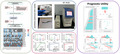

## 1 INTRODUCTION

Colorectal cancer (CRC) substantially influences cancer‐related death worldwide.^1^ Despite the recent advances in CRC management, the associated morbidity and mortality remain high.^2^ The last decade has witnessed a massive growth in our understanding of CRC genetic etiopathology.^3^ Identifying and highlighting such genetic contribution may help better understand the molecular basis of cancer patient prognosis with potential future targeted therapy.^4^

Noncoding RNAs have emerged as central genetic/epigenetic players in several cancers, including CRC.^5^, ^6^ The noncoding microRNAs (miRNAs) class has been implicated in CRC tumorigenesis/progression and treatment.^7^, ^8^ Indeed, their dysregulation may contribute to poor CRC outcomes, including metastasis and short survival.^9^, ^10^, ^11^

Our in silico analysis has revealed the microRNA‐497 (miR‐497) as one of the most iterated miRNAs in CRC, as will be detailed later on, and the B‐cell lymphoma 2 (*BCL2*) gene as one of its target genes that proved previously to play a central role in the regulation of apoptosis and was implicated in colorectal carcinogenesis, progression, and treatment resistance.^12^ Interestingly, previous studies have reported that miR‐497 can suppress proliferation and induce apoptosis via the Bcl2‐related molecular axis in several tissues and cancers, including neuronal cells, the “human umbilical vein endothelial cells,” breast cancer, and multiple myeloma.^13^, ^14^, ^15^ Zhu et al. found that miR‐497 could decrease the resistance to cisplatin in “human lung cancer cell lines” by targeting BCL2.^16^ Also, a recent study by Zheng et al. has proved that miR‐497/BCl2 axis could suppress cisplatin resistance in CRC cells.^17^ Nevertheless, no previous study demonstrated the impact of *BCL2*/miR‐497 expression ratio score on CRC prognosis and outcome. In this sense, the authors were interested in exploring the association of the *BCL2*/miR‐497 expression profile with the clinic‐pathological characteristics and outcomes of CRC patients to help their prognostic stratification and future individualized therapeutic management.

## 2 SUBJECTS AND METHODS

### 2.1 Bioinformatic selection of microRNA

Analysis of 2 TCGA datasets (TCGA‐COAD for colon adenocarcinoma and TCGA‐READ for rectal adenocarcinoma) from Genomic Data Commons Data Portal (https://portal.gdc.cancer.gov/) and 16 microarray public datasets (GSE2564, GSE10259, GSE38389, GSE18392, GSE30454, GSE35602, GSE38389, GSE33125, GSE49246, GSE35834, GSE54088, GSE41012, GSE41655, GSE48267, GSE73487, GSE77380) from Gene Expression Omnibus database (https://www.ncbi.nlm.nih.gov/geo/) revealed significant microRNAs in each comparison (Table 1). Log fold change and adjusted *p*‐values were identified for each experiment using the Database of Differentially Expressed miRNAs in Human Cancers (dbDEMC v3.0) (https://www.biosino.org/dbDEMC/index). The average fold change of microRNAs was estimated, the direction of expression across all studies was identified, and the total number of comparisons with significant expression was calculated. MiR‐497‐5p was selected because it was the most frequently downregulated microRNA across datasets. Functional enrichment analysis and gene targets of miR‐497‐5p identification in CRC KEGG pathway were identified using the DIANA‐miRPath v.3.0 (http://www.microrna.gr/miRPathv3); a “miRNA pathway analysis‐based webserver”.^18^

**TABLE 1:** Analyzed microRNA expression colorectal cancer experiments in public repositories (cancer versus normal tissues)

GEO ID	Sample case	Sample control	Number cases	Number controls	Up	Down
Colon cancer						
GSE2564	Colon tumor	Normal colon	10	5	3	4
GSE38389	Colon tumor	Normal colon	85	85	19	8
GSE18392	Colon tumor	Normal colon	116	29	157	153
GSE18392	Colon tumor TNM stage 2	Normal colon	44	29	147	153
GSE18392	Colon tumor TNM stage 3	Normal colon	38	29	134	137
GSE18392	Colon tumor TNM stage 4	Normal colon	15	29	82	103
GSE33125	Colon cancer	Normal colon	9	9	22	25
GSE49246	Colon cancer stage 2	Normal colon	40	40	407	437
GSE35834	Colon cancer	Normal colon	31	23	37	50
GSE48267	Colon cancer	Normal colon	61	61	44	53
GSE73487	Colon cancer	Normal tissue	64	23	0	9
GSE73487	Tubulovillous adenoma	Normal tissue	35	23	45	40
GSE73487	Serrated adenoma	Normal tissue	3	23	29	1
TCGA‐COAD	Colon adenocarcinoma	Normal tissue	441	8	158	181
Colorectal cancer						
GSE10259	Colorectal cancer	Normal colon	59	7	10	19
GSE10259	Colorectal cancer Duke A	Normal colon	5	8	14	9
GSE10259	Colorectal cancer Duke B	Normal colon	23	8	9	11
GSE10259	Colorectal cancer Duke C	Normal colon	20	8	25	27
GSE30454	Colorectal cancer	Normal colon	54	20	231	213
GSE30454	Hereditary nonpolyposis colon cancer	Normal colon	9	20	61	88
GSE30454	Lynch syndrome tumor	Normal colon	13	20	51	77
GSE35602	Colorectal cancer	Normal colon	17	4	3	19
GSE38389	Rectal tumor	Normal rectal mucosa	69	71	137	130
GSE54088	Colorectal cancer	Normal tissue	9	10	2	2
GSE41012	Colorectal cancer	Normal tissue	20	15	3	0
GSE41655	Colorectal adenocarcinoma	Normal colon	33	15	61	88
GSE41655	Colorectal adenoma	Normal colon	59	15	71	109
GSE77380	Rectum adenocarcinoma	Normal rectum	3	5	46	619
TCGA‐READ	Rectum adenocarcinoma	Normal tissue	158	3	147	174

Note

All experiments are microarray except the two TCGA datasets (microRNA sequencing). Up and down are the number of microRNAs found to be deregulated in the experiment.

### 2.2 Study population and tissue sampling

This retrospective study enrolled an eligible 53 pairs of “formalin‐fixed, paraffin‐embedded, FFPE” colorectal tissue samples archived in the Suez Canal University hospital pathology lab, Ismailia, Oncology Center of Mansoura Hospital, Mansoura, and El‐laban Pathology Laboratory, Port‐Said, Egypt, between January 2008 and December 2018. The inclusion criteria included archived paired primary CRC samples with no history of chemotherapy/radiotherapy before the surgery and availability of the related clinicopathological data from the medical records, including the survival follow‐up information. The stage system of the tumors was according to the International Union Against Cancer TNM staging system (8th ed.).^19^

**FIGURE 1:**
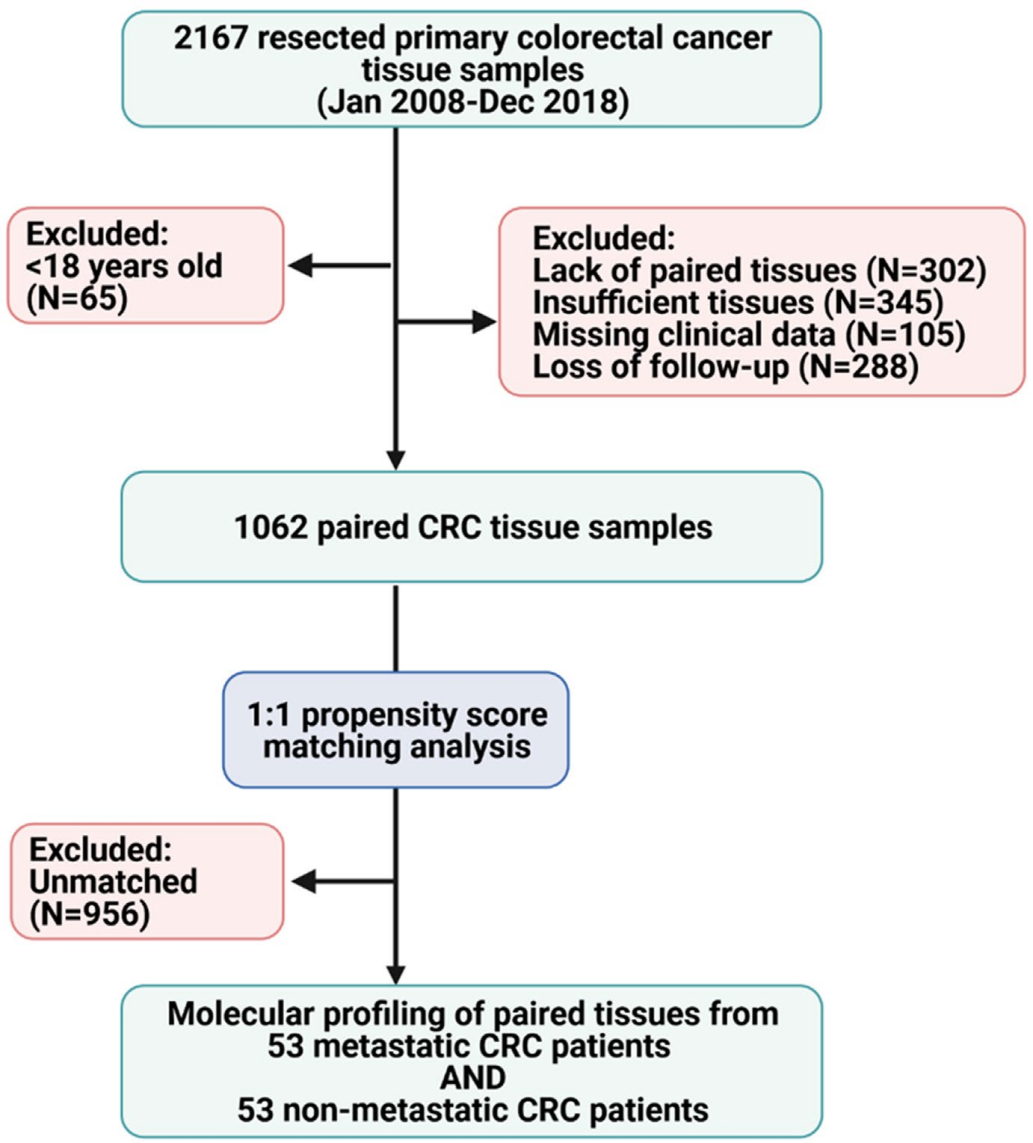
Workflow of the selection process. Screening of 2167 tissue specimens yielded 1062 with enough tissues and complete data. Propensity matching score analysis was performed to test the expression profile of the genes in matched metastatic and nonmetastatic cohorts after eliminating confounder bias

Samples with incomplete clinical and/or follow‐up data, history of receiving any therapeutic modality before resection, secondary CRC as well as samples without available paired noncancer tissue, tiny size tissue specimen available for molecular work, and those with low concentration or the extracted total RNA did not have enough quality to proceed in the downstream real‐time qPCR steps, were excluded as showed in Figure 1. The ethical/legal guidelines adopted by the Declaration of Helsinki were followed. The local Medical Research Ethics Committee granted ethical approval for this study, and the patient consent was waived as the enrolled samples in this retrospective study were archived.

### 2.3 Clinical assessment and follow‐up

Patient information was obtained from the medical records. These included patients’ demographic data, primary cancer site, pathology reports, and treatment modalities if available. Relapse, recurrence, further metastasis, and death reported during the follow‐up were reported. Overall survival was defined as the time from treatment to death (for any reason). Disease‐free survival represented the time from treatment to the recurrence (local, regional, distant) or death (for any reason). Survival times were categorized into short and prolonged times; short survival times were defined if ≤24 months after initial treatment.

### 2.4 Propensity scores matching analysis

The survival outcomes of metastatic and nonmetastatic colon cancer patients and the impact of transcriptomic signature of selected markers were compared *via* a propensity score matching analysis. This analysis was performed to adjust confounder variables using the *MatchIt* R package. The following covariates were adjusted: age, sex, obesity, tumor site, histopathological diagnosis, pathological grade, tumor size, lymph node metastasis, and lymphovascular invasion. Multivariate logistic regression was applied to create a balancing score as a distant measure for each patient. Next, metastatic and nonmetastatic cohorts were allocated using a one‐to‐one nearest neighbor algorithm without caliper adjustment to find pairs of patients that have the closest match in the two study groups. The quality of the matches in the two datasets (*N* = 53 patients in each group) were evaluated by estimating mean difference and average absolute standardized difference of covariates.^20^

### 2.5 *BCL2*/miR‐497 expression analysis

Total tissue RNA, including miRNAs, was isolated from the CRC samples using miRNeasy FFPE Kit (217504, Qiagen, Hilden, Germany) following the manufacturer's instructions. To ensure DNA‐free extracts, each sample was subjected to DNase I treatment (for 2 h at 37°C). RNA concentration/purity and integrity were tested by “NanoDrop ND‐1000 spectrophotometer (NanoDrop Tech., Inc.)” and “agarose gel electrophoresis,” respectively.

Reverse transcription (RT) for the total RNA was carried out by a high‐capacity complementary DNA RT kit (Applied Biosystems, P/N 4368814) in the case of *BCL2* gene expression quantification (assay ID Hs04986394_s1) compared to *GAPDH* gene (assay ID Hs02786624_g1). The RT reaction contains the RNA extract (5 μl), 100 mM of each dNTP (0.15 μl), “MultiScribe reverse‐transcriptase” (50 U/μl; 1 μl), 10 × RT buffer (1.5 μl), ribonuclease inhibitor (20 U/ml; 0.19 μl), gene‐specific TaqMan^®^ forward and reverse primers (3 μl of each) and nuclease‐free water (4.16 μl) was prepared for each RNA sample. For miR‐497 quantification, the total RNA was reverse transcribed using TaqMan MicroRNA RT kit (P/N 4366596; Thermo Fisher Scientific, Applied Biosystems) and either the miR‐497 specific stem‐loop primers (assay ID 001043) or the endogenous control RNU6B primers (assay ID 001093). The RT reactions of *BCL2* and miR‐497 were done on the “T‐Professional Basic, Biometra PCR System” (Biometra, Gottingen, Germany). Nontemplate and non‐RT enzyme negative controls were run with each experiment to exclude amplicon contamination. Then the quantitative Real‐Time PCR was carried out in duplicate in “StepOne Real‐Time PCR System” (Applied Biosystems) as described in detail previously.^10^, ^11^ All the steps of the qRT‐PCR were run following the “Minimum Information for Publication of Quantitative Real‐Time PCR Experiments (MIQE)” guidelines.^21^ The relative expression levels were calculated using the delta–delta CT (cyclic threshold) method.^22^

### 2.6 Statistical analysis

Relative expression levels of microRNA and genes were stratified by metastasis and plotted as box plots. Expression data were nonparametric; therefore, log transformation was employed. The Wilcoxon signed‐rank test was applied to compare cancer and its paired normal tissues, while the Mann–Whitney U test was carried out to test the difference between metastatic and nonmetastatic groups. To decipher the diagnostic accuracy of BCL2, miR‐497, and its ratio score, Receiver Operator Characteristic (ROC) curve analysis was performed, and area under the curve (AUC) was estimated for metastatic and nonmetastatic groups. Optimum cut‐off values with high sensitivity and specificity were identified. Univariate analysis was performed to identify variables influencing survival, followed by Cox regression analysis to identify independent risk factors for overall survival. Hazard ratio (HR) and 95% confidence interval (CI) were reported. Two‐sided *p*‐values <0.05 were regarded as significant. Spearman's correlation analysis was applied to identify the correlation between BCL2/miR‐497 ratio score and survival times. Kaplan–Meier curves were generated to compare patients with high‐ and low‐ratio scores based on the median value. Log‐Rank test with Benjamini and Hochberg adjustment for *p*‐value was applied. Under R version 4.0.5, *ggplot2* and *survminer* R packages were used for plotting. Finally, a Cox regression model was employed to construct a prognostic nomogram using *regplot* and *survival* R packages. Statistical analysis was performed using SPSS v27.0 (IBM Corp.), GraphPad prism v9.1.1 software (GraphPad, Inc.), and RStudio 1.4.1106 (R Foundation).

## 3 RESULTS

### 3.1 In silico data analysis

**FIGURE 2:**
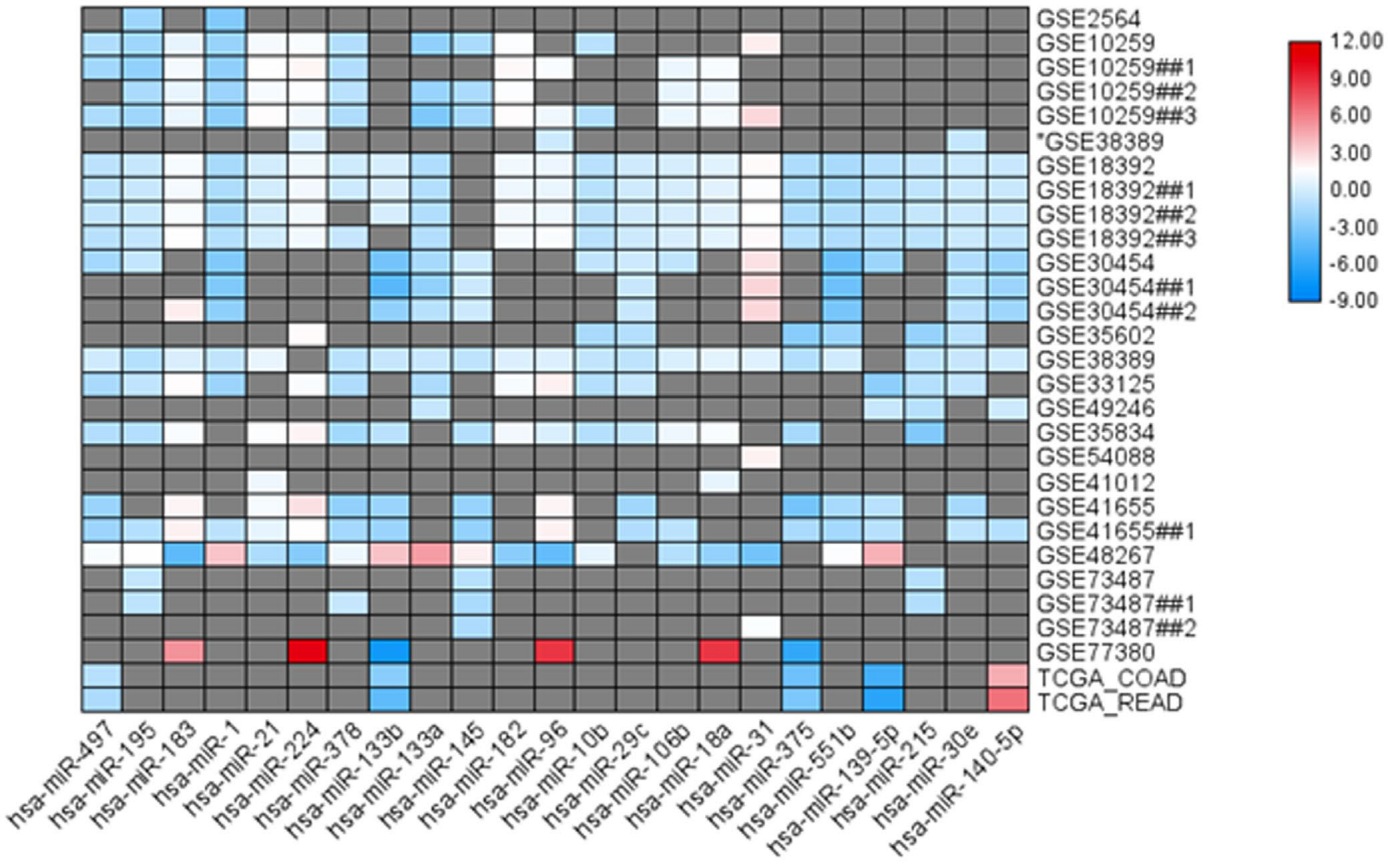
Most iterated significant microRNAs in the colon and colorectal cancer public datasets. Analysis of 29 datasets comparing cancer versus normal specimens in the colon and colorectal cancer patients showed miR‐497‐5p to be the most frequently downregulated microRNA

**FIGURE 3:**
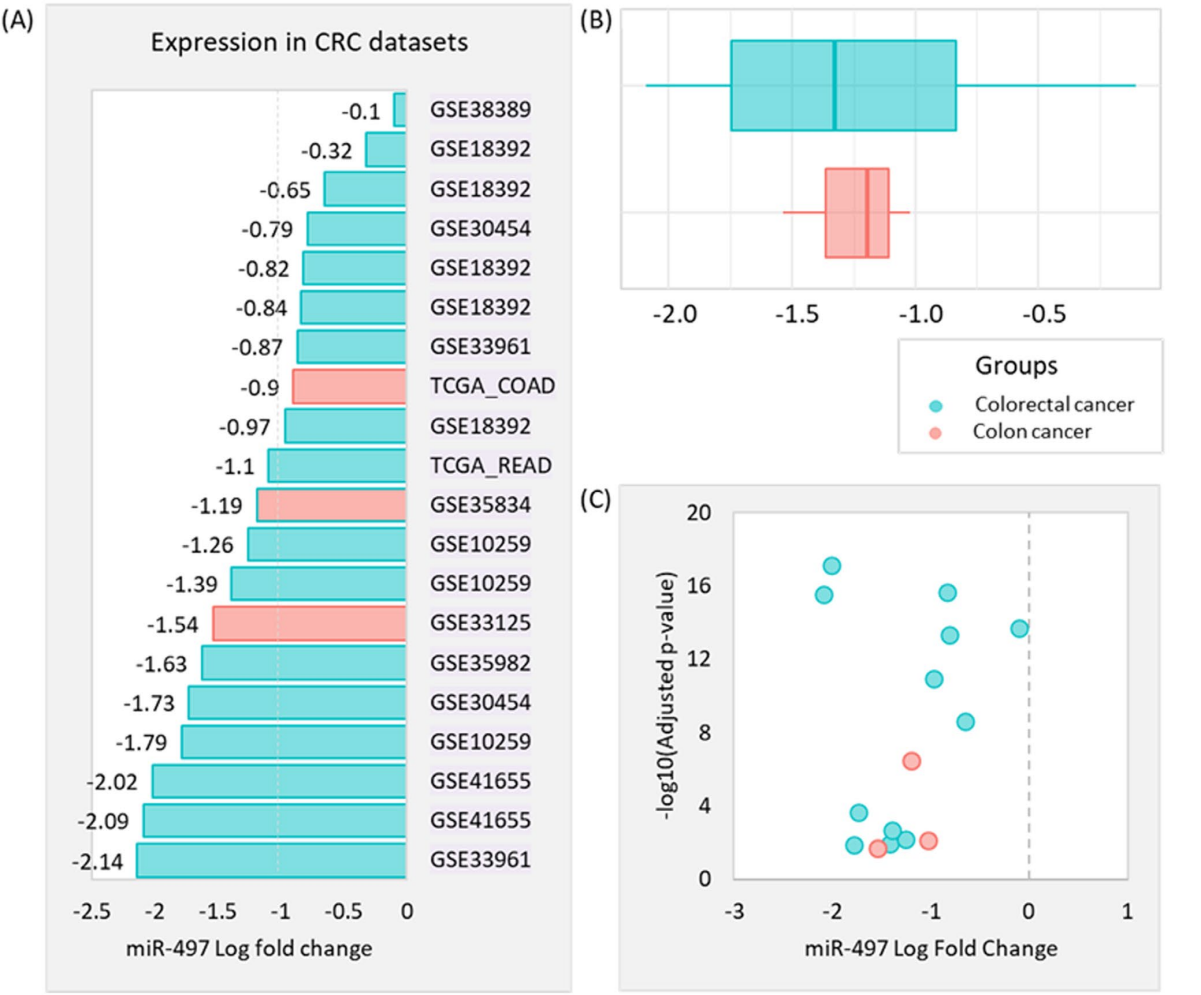
Downregulation of miR‐497‐5p in colorectal cancer datasets. The two TCGA datasets (TCGA‐COAD for colon adenocarcinoma and TCGA‐READ for rectal adenocarcinoma) were retrieved from Genomic Data Commons Data Portal (https://portal.gdc.cancer.gov/), while 16 microarray public datasets (GSE2564, GSE10259, GSE38389, GSE18392, GSE30454, GSE35602, GSE38389, GSE33125, GSE49246, GSE35834, GSE54088, GSE41012, GSE41655, GSE48267, GSE73487, GSE77380) were downloaded from Gene Expression Omnibus database (https://www.ncbi.nlm.nih.gov/geo/). (A) miR‐497 expression level in colorectal cancer patients of public datasets. (B) Box plot showing showed complete overlapping of the expression profile in the colon and colorectal cancers. (C) Volcano plot showing the correlation between fold change and corresponding *p*‐values

Analysis of 29 comparisons revealed a total of 2050 unique significant microRNAs in at least one analysis of CRC specimens. One of the most iterated microRNAs was miR‐497‐5p (Figure 2). It was downregulated in 16 different comparisons for cancer *versus* normal tissues (Figure 3). Similarly, the meta‐profiling of miR‐497 highlighted its putative tumor suppressor role in other types of cancers (Table S1). The expression level was the least in pancreatic cancer (GSE28955: FC = −4.69), sarcoma (GSE28423: FC = −4.49), and lymphoma (GSE45264: FC = −3.16). Lower miRNA expression was also noted in the circulation of the prostate (GSE31568: FC = −1.44) and renal (GSE38419: FC = −0.88) cancer patients. Furthermore, miR‐497 was two‐fold downregulated in tissues of CRC patients with poor outcomes (GSE33961: FC = −2.14) (Table S1).

### 3.2 Functional enrichment analysis

Pathway enrichment analysis revealed the involvement of miR‐497‐5p in cancer‐related pathways including proteoglycans in cancer (hsa05205|*p* = 1.45e‐11), hippo signaling pathway (hsa04390|*p* = 1.11e‐6), mTOR signaling pathway (hsa04150|*p* = 2.69e‐4), TGF‐beta signaling pathway (hsa04350|*p* = 7.64e‐4), and p53 signaling pathway (hsa00310|*p* = 8.27e‐4). In particular, miR‐497‐5p was significantly enriched in the CRC KEGG pathway [05210]. It has 12 gene targets: BRAF, BCL2, PIK3R2, SMAD3, BIRC5, AKT2, AKT3, CCD1, MAPK1, MAPK8, MAP2K1, MYC, and PIK3CA (Figure S1).

### 3.3 Baseline characteristics of the study population

The study population included 69 males and 37 females, 53.8% over 55 years old, and 62.3% were obese. Detailed information about clinical characteristics of propensity‐matched metastatic and nonmetastatic cohorts is described in Table 2. There were no significant differences in demographic and pathological features of both groups. However, a higher frequency of mortality was reported in 49.1% of metastatic cohorts compared to 18.9% in nonmetastatic cancer patients (*p* = 0.002). In addition, patients with metastasis at presentation showed shorter survival (*p* < 0.001), as expected.

**TABLE 2:** Characteristics of propensity score matched cohorts

Characteristics	Levels	Total (*N* = 106)	Nonmetastatic (*N* = 53)	Metastatic (*N* = 53)	*p*‐value
Demographic data					
Age (years)	≤55	49 (46.2)	27 (50.9)	22 (41.5)	0.43
	>55	57 (53.8)	26 (49.1)	31 (58.5)	
Sex	Female	37 (34.9)	18 (34)	19 (35.8)	0.83
	Male	69 (65.1)	35 (66)	34 (64.2)	
Obesity	Negative	40 (37.7)	23 (43.4)	17 (32.1)	0.31
	Positive	66 (62.3)	30 (56.6)	36 (67.9)	
Pathology data					
Location	Ascending	49 (46.2)	23 (43.4)	26 (49.1)	0.83
	Transverse	6 (5.7)	3 (5.7)	3 (5.7)	
	Descending	51 (48.1)	27 (50.9)	24 (45.3)	
Type	Adenocarcinoma	69 (65.1)	34 (64.2)	35 (66)	0.98
	Mucinous carcinoma	14 (13.2)	7 (13.2)	7 (13.2)	
	Signet ring carcinoma	14 (13.2)	7 (13.2)	7 (13.2)	
	Undifferentiated carcinoma	9 (8.5)	5 (9.4)	4 (7.5)	
Grade	Well‐differentiated	13 (12.3)	8 (15.1)	5 (9.4)	0.66
	Moderately differentiated	59 (55.7)	29 (54.7)	30 (56.6)	
	Poorly differentiated	34 (32.1)	16 (30.2)	18 (34)	
Tumor size stage	T1	12 (11.3)	6 (11.3)	6 (11.3)	0.21
	T2	49 (46.2)	25 (47.2)	24 (45.3)	
	T3	30 (28.3)	18 (34)	12 (22.6)	
	T4	15 (14.2)	4 (7.5)	11 (20.8)	
Lymph node stage	N0	45 (42.5)	24 (45.3)	21 (39.6)	0.11
	N1	43 (40.6)	24 (45.3)	19 (35.8)	
	N2	18 (17)	5 (9.4)	13 (24.5)	
Lymphovascular invasion	No	66 (62.3)	38 (71.7)	28 (52.8)	0.07
	Yes	40 (37.7)	15 (28.3)	25 (47.2)	
Outcomes					
Relapse	Negative	66 (62.3)	38 (71.7)	28 (52.8)	0.07
	Positive	40 (37.7)	15 (28.3)	25 (47.2)	
Mortality	Survived	70 (66)	43 (81.1)	27 (50.9)	**0.002**
	Died	36 (34)	10 (18.9)	26 (49.1)	
Disease‐free survival	Prolonged	84 (79.2)	51 (96.2)	33 (62.3)	**<0.001**
	Short	22 (20.8)	2 (3.8)	20 (37.7)	
Overall survival	Prolonged	90 (84.9)	52 (98.1)	38 (71.7)	**<0.001**
	Short	16 (15.1)	1 (1.9)	15 (28.3)	

Note

Data are presented as frequency and percentage. N: number. A two‐sided Chi‐square test was performed. Bold values indicate significant *p* < 0.05. Short survival was defined if ≤24 months after initial diagnosis.

### 3.4 Expression profile of miR‐497‐5p and *BCL2* in colon cancer tissues

**FIGURE 4:**
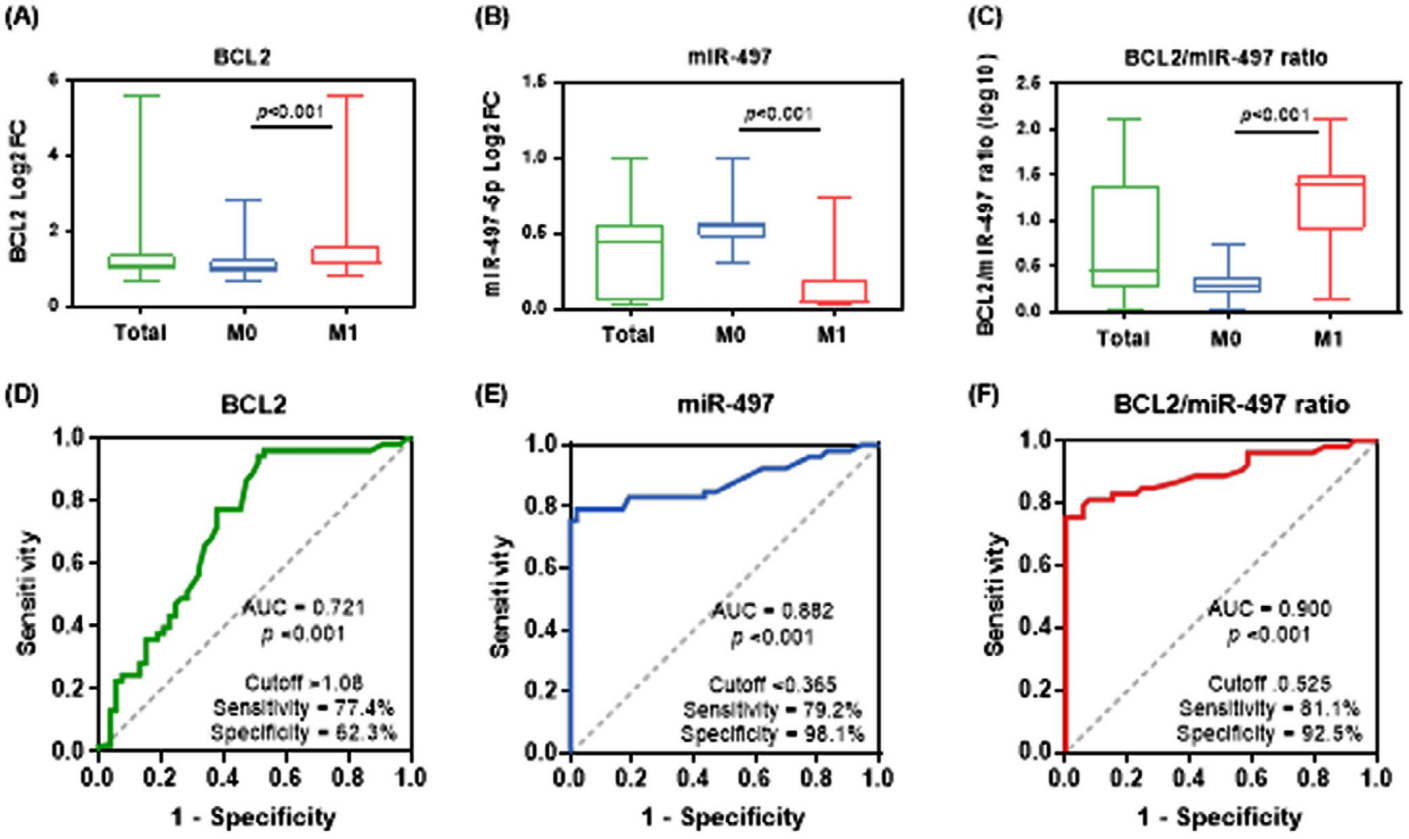
Relative expression profile of MIR‐497 and *BCL2* gene in colorectal cancer specimens. (A‐C) Data are shown as medians and quartiles. Being nonparametric, boxplot values were log‐transformed. The box defines upper and lower quartiles (25 and 75%, respectively), and the error bars indicate upper and lower adjacent limits. Fold change was normalized to RNU6B or *GAPDH* and calculated using the delta–delta CT method [=2^(−DDCT)^] compared to noncancer adjacent tissues. The Wilcoxon signed‐rank test was applied to compare cancer and its paired normal tissues, while the Mann–Whitney U test was carried out to test the difference between metastatic (M1) and nonmetastatic (M0) groups. (A) *BCL2* gene expression; (B) miR‐497‐5p expression; (C) *BCL2*/miR‐497 ratio risk score. (D–F) Receiver Operator Characteristics curve analysis showing the area under the curve (AUC) for predicting metastasis. The greater the area, the better the accuracy performance of the biomarker. (D) *BCL2* gene expression; (E) MIR‐497‐5p expression; (F) *BCL2*/MIR‐497 ratio risk score

B‐cell lymphoma‐2 gene was upregulated in metastatic samples (median = 1.16, 95%CI = 1.09–1.60) compared to nonmetastatic (median = 1.02, 95%CI = 0.89–1.25, *p* < 0.001). In contrast, lower levels of miR‐495‐5p were found in specimens with distant metastasis (median = 0.05, 95%CI = 0.04–0.20) than nonmetastatic samples (median = 0.54, 95%CI = 0.47–0.58, *p* < 0.001). Estimated ratio score between *BCL2* and miR‐497‐5p yielded a significant differential expression between the two cohort groups. Higher scores were noted in metastasis group (median = 1.39, 95%CI = 0.9–1.51) than nonmetastatic patients (median = 0.29, 95%CI = 0.19–0.39, *p* < 0.001) (Figure 4A‐C). ROC curve analysis showed *BCL2*/miR‐497 ratio score to have the highest predictive accuracy for metastasis at presentation. AUC was 0.90 (95%CI = 0.839–0.964, *p* < 0.001) at cut‐off of >0.525, with high sensitivity 81.1% (95%CI = 68.6%–89.4%) and specificity 92.5% (95%CI = 82.1%–97.0%) (Figure 4D‐F).

### 3.5 Prognostic value of miR‐497‐5p and *BCL2* in colon cancer

**FIGURE 5:**
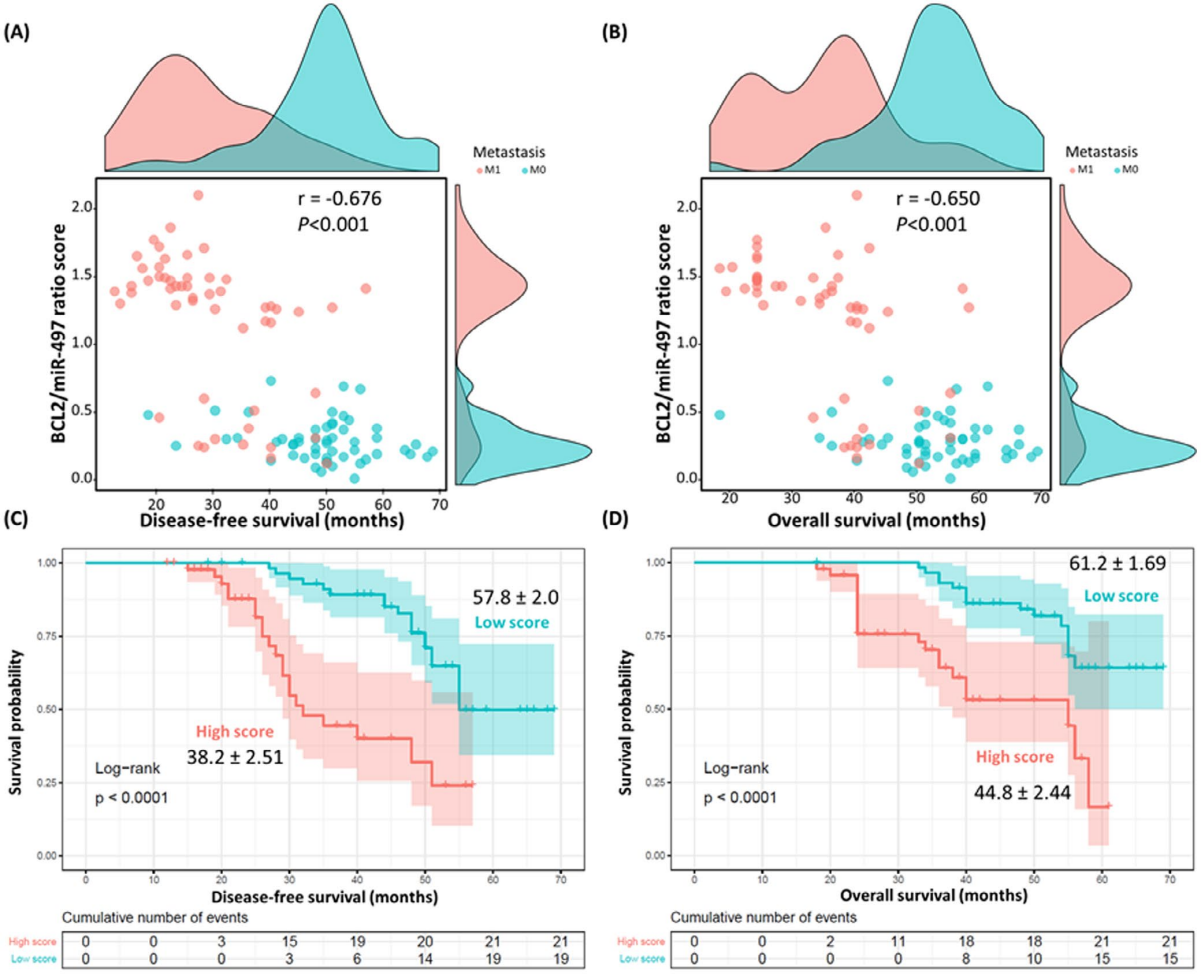
Prognostic value of *BCL2*/miR‐497 ratio score in colon cancer. (A) Correlation between ratio score and disease‐free survival. (B) Correlation between ratio score and overall survival. Patients with metastasis exhibited lower survival times. The distribution of patients showed two clusters: one cluster (upper left corner) composed of metastatic patients with remarkable high ratio score and most of them showed low survival times of less than 45 months, and the other cluster (lower right corner) included cases of nonmetastasis mixed with few metastatic samples showing lower survival rates for M1 cases. Marginal histogram plots showed the density of cases stratified by metastasis: M1 (red) and M0 (green). Rho coefficient (*r*) of Spearman's correlation analysis showed a moderate negative correlation between the ratio score and survival times. (C) Kaplan–Meier survival curve for disease‐free survival analysis. (D) Kaplan–Meier survival curve for overall survival analysis. Log Rank (Mantel Hanzel) test was used. Patients were categorized according to the median value. Patients with high scores showed lower overall and disease‐free survival rates

**FIGURE 6:**
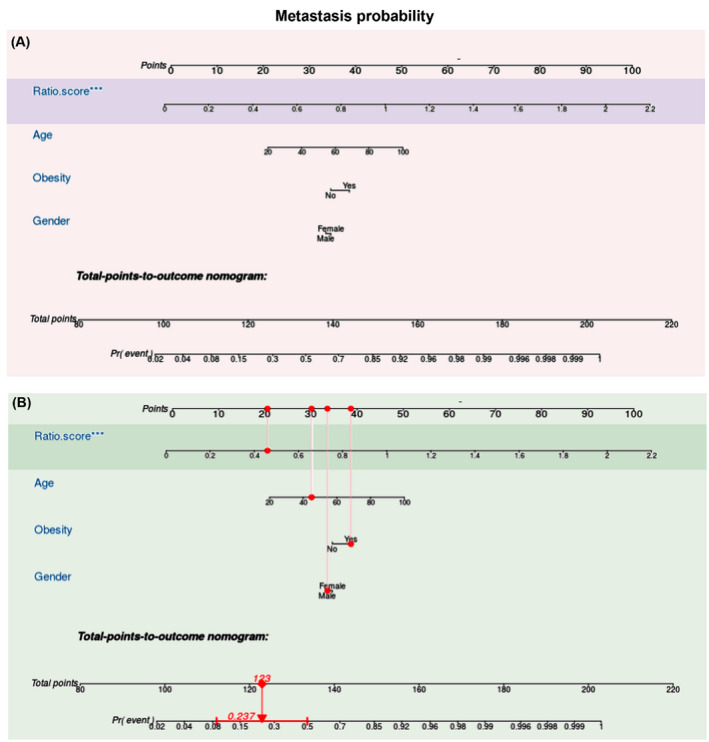
Nomogram for predicting metastasis. (A) The nomogram was constructed based on demographic features of patients and ratio risk score. The outcome measured was metastasis. The logistic regression model was applied. (B) Example for using the nomogram. Assumed having a 45‐year‐old obese male patient whose tissue microRNA risk score was high at 0.45. Each variable will be scored on its points scale. The scores for all variables are added to obtain the total score “of 123,” and a vertical line is drawn from the total points’ row to estimate the probability of metastasis “23.7%”

Table 3 demonstrated the association between the expression levels and demographic, clinical, and pathological parameters. Tested genes were significantly associated with metastasis, clinical stage, and mortality. In univariate analysis, expired patients were three to four times more likely to be obese (80.6% versus 52.9%, *p* = 0.006), have metastasis at presentation (72.2% vs. 38.6%, *p* = 0.002), have lymphovascular invasion (55.6% vs. 28.6%, *p* = 0.011), and have higher ratio score (66.7% vs. 41.4%, *p* = 0.023). Cox regression model revealed that high‐risk score was nearly three times more likely to die (HR = 2.82, 95%CI = 1.22–6.55) (Table 4). The ratio score was negatively correlated with disease‐free survival (*r* = −0.676, *p* < 0.001) and overall survival times (*r* = −0.650, *p* < 0.001). Patients with metastasis exhibited lower survival times (Figure 5A‐B). When patients were categorized according to the median ratio score into high‐score and low‐score groups, Kaplan–Meier curves showed lower survival rates in cohorts with high‐score compared to low‐score patients (Figure 5C‐D). A prognostic nomogram to predict metastasis at presentation was generated using the ratio score with demographic characteristics of patients, which showed good agreement with the actual outcome (Figure 6).

**TABLE 3:** Univariate association analysis of MIR‐497 and *BCL2* expression with clinic‐pathological features

Characteristics		No. of cases	BCL2 log2FC	*p*‐value	miR−497 log2FC	*p*‐value	Log10 ratio score	*p*‐value
Age (years)	≤55	49 (46.2)	1.12 (0.95–1.28)	0.28	0.45 (0.05–0.56)	0.75	0.4 (0.28–1.33)	0.90
	>55	57 (53.8)	1.13 (1.01–1.52)		0.44 (0.05–0.56)		0.52 (0.25–1.42)	
Sex	F	37 (34.9)	1.11 (0.97–1.45)	0.97	0.48 (0.04–0.57)	0.84	0.43 (0.26–1.45)	0.86
	M	69 (65.1)	1.13 (0.98–1.36)		0.45 (0.05–0.56)		0.46 (0.27–1.38)	
Obesity	Negative	40 (37.7)	1.07 (0.95–1.34)	0.44	0.45 (0.06–0.56)	0.89	0.4 (0.25–1.32)	0.52
	Positive	66 (62.3)	1.14 (1.03–1.42)		0.45 (0.05–0.56)		0.5 (0.28–1.42)	
Location	Ascending	49 (46.2)	1.13 (0.96–1.48)	0.66	0.44 (0.05–0.55)	0.49	0.52 (0.27–1.44)	0.58
	Transverse	6 (5.7)	1.21 (0.95–1.61)		0.56 (0.05–0.67)		0.36 (0.2–1.35)	
	Descending	51 (48.1)	1.11 (0.98–1.29)		0.45 (0.06–0.57)		0.4 (0.27–1.31)	
Type	Adenocarcinoma	69 (65.1)	1.11 (0.94–1.33)	0.29	0.45 (0.05–0.56)	0.62	0.4 (0.25–1.44)	0.86
	Mucinous	14 (13.2)	1.24 (1.01–1.68)		0.52 (0.06–0.58)		0.61 (0.28–1.31)	
	Signet ring	14 (13.2)	1.13 (1.06–1.28)		0.44 (0.07–0.68)		0.47 (0.22–1.21)	
	Undifferentiated	9 (8.5)	1.13 (1.07–1.82)		0.5 (0.06–0.57)		0.49 (0.32–1.29)	
Grade	G1	13 (12.3)	1.13 (0.88–1.34)	0.23	0.44 (0.04–0.51)	0.24	0.41 (0.23–1.42)	0.89
	G2/3	93 (87.7)	1.12 (0.98–1.46)		0.46 (0.05–0.56)		0.46 (0.27–1.35)	
Tumor size	T1/2	61 (57.5)	1.13 (0.95–1.34)	0.53	0.45 (0.05–0.56)	0.72	0.4 (0.27–1.41)	0.81
	T3/4	45 (42.5)	1.12 (1.01–1.57)		0.45 (0.05–0.57)		0.66 (0.23–1.38)	
LN invasion	Negative	66 (62.3)	1.11 (0.97–1.4)	0.76	0.5 (0.06–0.58)	**0.033**	0.33 (0.21–1.3)	0.08
	Positive	40 (37.7)	1.13 (0.98–1.38)		0.39 (0.05–0.55)		0.53 (0.29–1.42)	
Metastasis	Negative	53 (50)	1.02 (0.9–1.25)	**<0.001**	0.54 (0.47–0.58)	**<0.001**	0.29 (0.2–0.4)	**<0.001**
	Positive	53 (50)	1.16 (1.09–1.6)		0.05 (0.04–0.2)		1.39 (0.9–1.51)	
Site of metastasis	Liver	44 (83)	1.15 (1.06–1.51)	0.12	0.05 (0.04–0.27)	0.75	1.35 (0.78–1.51)	0.53
	Lung	9 (17)	1.33 (1.15–2.12)		0.05 (0.04–0.2)		1.41 (0.96–1.59)	
LVI	Negative	66 (62.3)	1.13 (0.97–1.59)	0.21	0.47 (0.06–0.56)	0.13	0.43 (0.26–1.37)	0.71
	Positive	40 (37.7)	1.12 (0.97–1.32)		0.39 (0.05–0.55)		0.5 (0.27–1.41)	
Dukes	A/B	24 (22.6)	1.04 (0.9–1.2)	**0.026**	0.54 (0.49–0.6)	**<0.001**	0.28 (0.19–0.38)	**<0.001**
	C/D	82 (77.4)	1.14 (1.02–1.43)		0.34 (0.05–0.55)		0.72 (0.29–1.45)	
Relapse	Negative	66 (62.3)	1.09 (0.95–1.39)	0.17	0.47 (0.06–0.57)	0.21	0.42 (0.23–1.31)	0.18
	Positive	40 (37.7)	1.15 (1.03–1.43)		0.41 (0.05–0.54)		0.57 (0.3–1.44)	
Died	Negative	70 (66)	1.07 (0.94–1.26)	**0.008**	0.48 (0.06–0.57)	**0.038**	0.33 (0.23–1.29)	**0.004**
	Positive	36 (34)	1.28 (1.06–1.59)		0.2 (0.04–0.54)		0.99 (0.33–1.49)	
Short DFS	Negative	84 (79.2)	1.07 (0.95–1.29)	**<0.001**	0.49 (0.13–0.57)	**<0.001**	0.33 (0.23–1.04)	**<0.001**
	Positive	22 (20.8)	1.4 (1.15–1.88)		0.04 (0.04–0.06)		1.47 (1.38–1.6)	
Short OS	Negative	90 (84.9)	1.07 (0.95–1.28)	**<0.001**	0.49 (0.06–0.57)	**<0.001**	0.33 (0.24–1.21)	**<0.001**
	Positive	16 (15.1)	1.42 (1.28–1.89)		0.04 (0.04–0.05)		1.51 (1.43–1.63)	

Note

The expression level is shown as median (quartiles). Mann–Whitney U test was used. Bold values indicate significant *p* < 0.05. LN: lymph node; LVI: Lymph‐vascular invasion; DFS: disease‐free survival; OS: overall survival.

**TABLE 4:** Characteristics of colon cancer patients according to survival

Characteristics	Levels	Survived (*N* = 70)	Died (*N* = 36)	*p*‐value	HR (95%CI)
Age (years)	≤55	33 (47.1)	16 (44.4)	0.83	*Reference*
	>55	37 (52.9)	20 (55.6)		1.11 (0.49–2.50)
Sex	Female	22 (31.4)	15 (41.7)	0.39	*Reference*
	Male	48 (68.6)	21 (58.3)		0.64 (0.27–1.47)
Obesity	Negative	33 (47.1)	7 (19.4)	**0.006**	*Reference*
	Positive	37 (52.9)	29 (80.6)		3.69 (1.43–9.54)
Location	Ascending	32 (45.7)	17 (47.2)	0.65	*Reference*
	Transverse	3 (4.3)	3 (8.3)		1.88 (0.34–10.3)
	Descending	35 (50)	16 (44.4)		0.86 (0.37–1.98)
Type	Adenocarcinoma	45 (64.3)	24 (66.7)	0.69	*Reference*
	Mucinous carcinoma	11 (15.7)	3 (8.3)		0.51 (0.13–2.01)
	Signet ring carcinoma	9 (12.9)	5 (13.9)		1.04 (0.31–3.45)
	Undifferentiated carcinoma	5 (7.1)	4 (11.1)		1.50 (0.36–6.11)
Grade	G1	32 (88.9)	32 (88.9)	0.79	*Reference*
	G2/3	32 (88.9)	32 (88.9)		1.18 (0.33–4.13)
Tumor size stage	T1/2	37 (52.9)	24 (66.7)	0.21	*Reference*
	T3/4	33 (47.1)	12 (33.3)		0.56 (0.24–1.29)
LN invasion	Negative	28 (40)	17 (47.2)	0.53	*Reference*
	Positive	42 (60)	19 (52.8)		0.74 (0.33–1.67)
Metastasis	Negative	43 (61.4)	10 (27.8)	**0.002**	*Reference*
	Positive	27 (38.6)	26 (72.2)		4.14 (1.72–9.92)
LVI	Negative	50 (71.4)	16 (44.4)	**0.011**	*Reference*
	Positive	20 (28.6)	20 (55.6)		3.12 (1.35–7.21)
Duke stage	A/B	20 (28.6)	4 (11.1)	**0.042**	*Reference*
	C/D	50 (71.4)	32 (88.9)		3.2 (1.0–10.2)
Ratio score	Low score	41 (58.6)	12 (33.3)	**0.023**	*Reference*
	High score	29 (41.4)	24 (66.7)		2.82 (1.22–6.55)

Note

Data are presented as frequency and percentage. A two‐sided Chi‐square test was performed. *P*‐value less than 0.05 was set to be significant (bold values). Univariate Cox regression analysis was performed and shown in the last column. Hazard ratio (HR) and 95% confidence intervals (CI) are reported. Log10 Ratio score at >0.45 (median value) was set as a high score, based on ROC curve analysis. N: number; LN: lymph node; LVI: Lymph‐vascular invasion.

## 4 DISCUSSION

CRC's tendency to invasion/metastasis is one of the major factors leading to poor prognosis.^23^ Identifying new genetic/epigenetic biomarkers associated with CRC metastasis and survival could help improve cancer management.^24^

In this work, we explored the association of *BCL2*, miR‐479, and *BCL2*/miR‐479 ratio with poor prognosis in terms of metastasis and short survival in patients with CRC. We found that *BCL2* was upregulated in metastatic samples compared to nonmetastatic ones. In contrast, miR‐495‐5p downregulation was found in specimens with distant metastasis than nonmetastatic samples. The estimated ratio score between *BCL2* and miR‐497‐5p yielded a significant differential expression between the two cohort groups. Also, ROC curve analysis showed *BCL2*/miR‐497 ratio score to have the highest predictive accuracy for metastasis at presentation. Furthermore, the ratio score showed a negative correlation with disease‐free survival and overall survival, as well as included in a newly generated prognostic nomogram to predict metastasis, among other parameters. These results are consistent with previous studies that reported the implication of *BCL2* and miR‐497 in cancer, including the CRC,^12^, ^25^, ^26^, ^27^ and support that analyzing combined markers is better than an individual molecule in cancer diagnostics and/or prognostication.^28^

The pro‐survival BCL2 is one of the “anti‐apoptotic BCL2 family proteins” implicated in promoting cancer cell proliferation, metastatic spread, and resistance to anticancer therapy.^29^ Several mechanisms have been proposed to explain the *BCL2* gene overexpression, including increasing the rate of gene transcription,^30^ gene amplification (increased gene copy number),^31^ chromosomal translocations,^32^ and posttranscriptional–translational modifications that augment the prosurvival activity of the specified proteins.^33^, ^34^, ^35^ Accumulating evidence proved that deregulated BCL2 family expression is not provided to occur only in the tumorigenesis stage of cancer but can be observed in all stages of cancer progression, including metastasis and even in the anticancer therapeutic resistance stage.^36^, ^37^, ^38^ A meta‐analysis of 40 articles showed a significant association of BCL2 expression with pathological grade, clinical stage, overall, and disease‐free survival in patients with CRC.^39^ Bcl‐2 has been shown to prolong cell survival by inhibiting apoptosis.^40^, ^41^ Abnormal activation of the *Bcl*‐*2* gene appears to be an early event in colorectal tumorigenesis.^42^ It is worth noting that cancer development and progression rely on the overexpression of antiapoptotic gene players and underexpression of the proapoptotic ones. The outcome of the interplay between these signatures varies according to the cancer type and even could be different within the same cancer type.^43^, ^44^ This could partly explain the heterogeneity/controversy between the observed prognostic signature of *BCL2* in different cancer types, including CRC, in the present study and previous reports.

MiR‐497 dysregulation reflects a complex network that is influenced by several factors.^27^ Interestingly, miR‐497 downregulation in this study agrees with many independent online gene expression omnibus (GEO) experiments, including the GSE41655 (https://www.ncbi.nlm. nih.gov/geo/query/acc.cgi?acc = GSE41655), GSE35834,^45^ and GSE68204,^46^ among others in which miR‐497 downregulation was observed in CRC tissues compared to the adjacent noncancerous mucosa (all *p* < 0.001). Additionally, several previous studies have uncovered the molecular role(s) by which miR‐497 can impact CRC tumorigenesis and/or progression. For example, Guo et al. reported that miR‐497 downregulation could upregulate “insulin‐like growth factor 1 receptor” with subsequent increase of “PI3K/Akt” signaling, contributing to the malignant behavior of CRC cells.^47^ Zhang et al. also explored miR‐497 overexpression can reduce the ability of CRC cells to invade tissues, and this inhibition was mediated through “Fos‐related‐antigen‐1” regulation.^48^ Similarly, Xu et al. reported that miR‐497 targeted upregulation in CRC tissues can suppress the proliferation and migration/invasion of CRC cells by “insulin receptor substrate‐1” degradation.^49^ Ectopic miR‐497 expression induced by Wang et al. was found to suppress the CRC cell oncogenic hallmarks and augment the sensitivity of these cells to the chemotherapeutic agents via “kinase suppressor of Ras‐1” oncogene regulation.^50^ Also, Zou et al. concluded the same miR‐497 downregulated signature in patients with CRC, but in the sera of patients, which was an independent parameter for CRC.^26^ These findings supported the potential suppressor role of miR‐497 that plays in CRC.

Some limitations should be addressed in this study. The sample size of eligible cohorts was considerably small; thus, multivariate analysis including many confounders was challenging. However, propensity matching in nature reduces the bias of confounding variables and mimics randomization leading to analysis of balanced groups. To the best of our knowledge, our study shows for the first time the relationship between the two study molecules in a group of CRC patients with metastasis and nonmetastasis.

## 5 CONCLUSION

In summary, our findings in this study suggest the essential role of the *BCL2*/miR‐497 ratio as a prognostic ratio for CRC in terms of association with metastasis and poor survival indices. However, it is worth noting that our study lacks the functional studies that prove the exact mechanism by which miR‐497 and its molecular target BCL2 could play in CRC samples. Thus, future studies to assess the exact mechanistic roles of the BCL2/miR‐497 ratio in vivo and clinical context are warranted. The present findings could have important implications for the prognosis of patients with CRC and could be assigned in future anticancer therapeutic management protocols.

## CONFLICT OF INTEREST

The authors report no conflicts of interest.

## AUTHOR CONTRIBUTION

All authors contributed to data analysis, drafting, or revising the article, have agreed on the journal to which the article has been submitted, gave final approval of the version to be published, and agree to be accountable for all aspects of the work.

## Supporting information

Supplementary Material

## Data Availability

All data generated or analyzed during this study are included in this submitted article and supplementary materials.
