# Supplementary material for: Association of B‐cell lymphoma 2/microRNA‐497 gene expression ratio score with metastasis in patients with colorectal cancer: A propensity‐matched cohort analysis
Source: J Clin Lab Anal. 2022 Jan 7;36(2):e24227. doi: 10.1002/jcla.24227 (PMC8841134; doi:10.1002/jcla.24227)
Supplement: Supplementary file 1 — Supplementary Material [file JCLA-36-e24227-s001.docx]

**Original Article**

**Association of B-cell lymphoma 2/microRNA-497 gene expression ratio score with metastasis in patients with colorectal cancer: A propensity-matched cohorts' analysis**

**Table S1.** Diagnostic and prognostic value of miR-497 in different types of cancer

| **GEO ID** | **Cancer type** | **Comparison** | **LogFC** |
| --- | --- | --- | --- |
| GSE28955 | Pancreatic cancer | Cancer vs normal | -4.69 |
| GSE28423 | Sarcoma | Cancer vs normal | -4.49 |
| GSE45264 | Lymphoma | Cancer vs normal | -3.16 |
| GSE40355 | Bladder cancer | Cancer vs normal | -3.13 |
| GSE19945 | Lung cancer | Cancer vs normal | -2.92 |
| GSE48088 | Breast cancer | Cancer vs normal | -2.86 |
| GSE51853 | Lung cancer | Cancer vs normal | -2.72 |
| GSE74190 | Lung cancer | Cancer vs normal | -2.45 |
| GSE11016 | Kidney cancer | Cancer vs normal | -2.26 |
| TCGA_CESC | Cervical cancer | Cancer vs normal | -2.22 |
| GSE11016 | Kidney cancer | Cancer vs normal | -2.15 |
| GSE33961 | Colorectal cancer | Poor outcome vs good outcome | -2.14 |
| GSE51853 | Lung cancer | Cancer vs normal | -2.12 |
| GSE41655 | Colorectal cancer | Cancer vs normal | -2.09 |
| GSE11016 | Kidney cancer | Cancer vs normal | -2.05 |
| GSE45666 | Breast cancer | Cancer vs normal | -2.02 |
| GSE41655 | Colorectal cancer | Cancer vs normal | -2.02 |
| GSE19945 | Lung cancer | Cancer vs normal | -1.99 |
| GSE61438 | Breast cancer | Cancer vs normal | -1.96 |
| GSE45666 | Breast cancer | Cancer vs normal | -1.91 |
| GSE19945 | Lung cancer | Cancer vs normal | -1.9 |
| GSE45666 | Breast cancer | Cancer vs normal | -1.9 |
| TCGA_LUSC | Lung cancer | Cancer vs normal | -1.84 |
| GSE19945 | Lung cancer | Cancer vs normal | -1.8 |
| GSE10259 | Colorectal cancer | Cancer vs normal | -1.79 |
| GSE26595 | Gastric cancer | Cancer vs normal | -1.78 |
| GSE45666 | Breast cancer | Cancer vs normal | -1.78 |
| TCGA_UCEC | Endometrial cancer | Cancer vs normal | -1.78 |
| GSE30454 | Colorectal cancer | Cancer vs normal | -1.73 |
| GSE38167 | Breast cancer | Cancer vs normal | -1.73 |
| GSE45666 | Breast cancer | Cancer vs normal | -1.71 |
| GSE24996 | Melanoma | Cancer vs normal | -1.7 |
| GSE35982 | Colorectal cancer | Cancer vs normal | -1.63 |
| GSE39486 | Brain cancer | Cancer vs normal | -1.62 |
| GSE35579 | Melanoma | Cancer vs normal | -1.6 |
| GSE29742 | Neuroendocrine neoplasia | Cancer vs normal | -1.58 |
| GSE33125 | Colon cancer | Cancer vs normal | -1.54 |
| GSE44124 | Breast cancer | Cancer vs normal | -1.54 |
| GSE74190 | Lung cancer | Cancer vs normal | -1.54 |
| GSE45666 | Breast cancer | Cancer vs normal | -1.5 |
| GSE45666 | Breast cancer | Cancer vs normal | -1.46 |
| GSE40525 | Breast cancer | Cancer vs normal | -1.44 |
| GSE31568 | Prostate cancer | Blood | -1.44 |
| GSE25820 | Pancreatic cancer | Cancer vs normal | -1.42 |
| GSE10259 | Colorectal cancer | Cancer vs normal | -1.39 |
| GSE40525 | Breast cancer | Cancer vs normal | -1.35 |
| GSE53870 | Biliary tract cancer | Cancer vs normal | -1.34 |
| GSE51853 | Lung cancer | Cancer vs normal | -1.33 |
| GSE10259 | Colorectal cancer | Cancer vs normal | -1.26 |
| TCGA_BLCA | Bladder cancer | Cancer vs normal | -1.26 |
| GSE74562 | Pancreatic cancer (PANC-1) | Treatment | -1.25 |
| GSE6188 | Esophageal cancer | Cancer vs normal | -1.24 |
| GSE40525 | Breast cancer | Cancer vs normal | -1.24 |
| TCGA_KICH | Kidney cancer | Cancer vs normal | -1.24 |
| TCGA_STAD | Gastric cancer | Cancer vs normal | -1.21 |
| GSE74190 | Lung cancer | Cancer vs normal | -1.2 |
| GSE35834 | Colon cancer | Cancer vs normal | -1.19 |
| TCGA_READ | Colorectal cancer | Cancer vs normal | -1.1 |
| TCGA_KIRP | Kidney cancer | Cancer vs normal | -1.09 |
| TCGA_LIHC | Hepatocellular carcinoma | Cancer vs normal | -1.09 |
| GSE40525 | Breast cancer | Cancer vs normal | -1.05 |
| TCGA_HNSC | Head and neck cancer | Cancer vs normal | -1.05 |
| GSE63805 | Lung cancer | Cancer vs normal | -0.98 |
| GSE18392 | Colon cancer | Cancer vs normal | -0.97 |
| GSE36915 | Hepatocellular carcinoma | Cancer vs normal | -0.93 |
| GSE36682 | Nasopharyngeal cancer | Cancer vs normal | -0.92 |
| GSE40525 | Breast cancer | Cancer vs normal | -0.91 |
| TCGA_COAD | Colon cancer | Cancer vs normal | -0.9 |
| GSE38419 | Kidney cancer | Blood | -0.88 |
| TCGA_ESCA | Esophageal cancer | Cancer vs normal | -0.88 |
| GSE33961 | Colorectal cancer | Poor outcome vs good outcome | -0.87 |
| GSE45666 | Breast cancer | High grade vs low grade | -0.87 |
| GSE45238 | Oral squamous cell carcinoma | Cancer vs normal | -0.87 |
| GSE18392 | Colon cancer | Cancer vs normal | -0.84 |
| GSE28700 | Gastric cancer | Cancer vs normal | -0.84 |
| GSE18392 | Colon cancer | Cancer vs normal | -0.82 |
| GSE21362 | Hepatocellular carcinoma | Cancer vs normal | -0.81 |
| GSE30454 | Colorectal cancer | Cancer vs normal | -0.79 |
| GSE40525 | Breast cancer | Cancer vs normal | -0.79 |
| GSE51853 | Lung cancer | Cancer vs normal | -0.79 |
| GSE33232 | Head and neck cancer | Cancer vs normal | -0.78 |
| GSE40525 | Breast cancer | Cancer vs normal | -0.77 |
| GSE40525 | Breast cancer | Cancer vs normal | -0.77 |
| GSE18392 | Colon cancer | Cancer vs normal | -0.65 |
| GSE29135 | Lung cancer | Subtype1 vs substype2 | -0.62 |
| TCGA_THCA | Thyroid cancer | Cancer vs normal | -0.62 |
| TCGA_LUAD | Lung cancer | Cancer vs normal | -0.57 |
| GSE32960 | Nasopharyngeal cancer | Cancer vs normal | -0.56 |
| TCGA_BRCA | Breast cancer | Cancer vs normal | -0.41 |
| GSE40807 | Thyroid cancer | Cancer vs normal | -0.4 |
| GSE18392 | Colon cancer | High grade vs low grade | -0.32 |
| TCGA_UCEC | Endometrial cancer | Cancer vs normal | -0.32 |
| GSE10694 | Hepatocellular carcinoma | Cancer vs normal | -0.28 |
| GSE10694 | Hepatocellular carcinoma | Cancer vs normal | -0.26 |
| TCGA_TGCT | Testicular cancer | High grade vs low grade | -0.25 |
| GSE30656 | Cervical cancer | High grade vs low grade | -0.24 |
| GSE31377 | Lymphoma | Cancer vs normal | -0.23 |
| GSE15008 | Lung cancer | Cancer vs normal | -0.16 |
| TCGA_ESCA | Esophageal cancer | Cancer vs normal | -0.13 |
| GSE37407 | Breast cancer | Metastasis | -0.12 |
| TCGA_PRAD | Prostate cancer | Cancer vs normal | -0.11 |
| TCGA_STAD | Gastric cancer | Cancer vs normal | -0.11 |
| GSE16025 | Lung cancer | Cancer vs normal | -0.1 |
| GSE38389 | Colorectal cancer | Cancer vs normal | -0.1 |
| GSE16025 | Lung cancer | Cancer vs normal | -0.09 |

Data source: dbDEMC (<https://www.biosino.org/dbDEMC/index>).

**
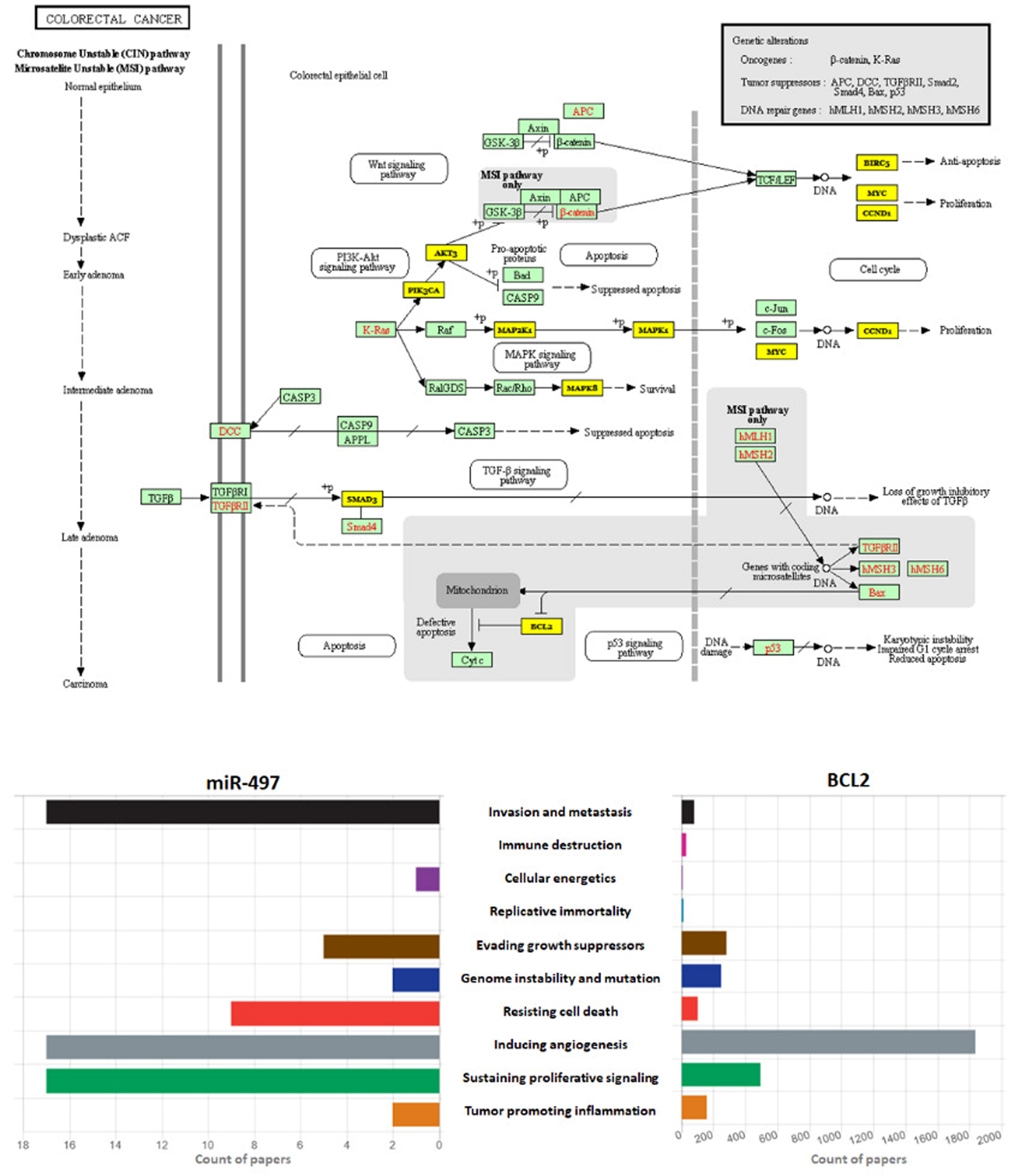
**

**Figure S1. MicroRNA-497 and colorectal cancer pathway and hallmarks.** The top KEGG pathway (ID: 05210) shows that miR-497 targets cancer-related genes (yellow boxes) in the CRC pathway. These interactions are validated experimentally by luciferase reporter assay, photoactivatable ribonucleoside-enhanced crosslinking and immunoprecipitation, or High-throughput sequencing of RNA isolated by crosslinking immunoprecipitation [Data source: “<http://diana.imis.athena-innovation.gr/>”]. The lower panel shows the role of BCL2 and miR-497 in cancer hallmarks derived from the “Cancer Hallmarks Analytics Tool (CHAT)” web browser, which depends on the analysis of cancer in the literature based on a text-mining analysis of 26 million PubMed abstracts [Data source: “http://chat.lionproject.net”].
